# Mitochondrial DNA copy number augments performance of A_1_C and oral glucose tolerance testing in the prediction of type 2 diabetes

**DOI:** 10.1038/srep43203

**Published:** 2017-03-02

**Authors:** Seong Beom Cho, InSong Koh, Hye-Young Nam, Jae-Pil Jeon, Hong Kyu Lee, Bok-Ghee Han

**Affiliations:** 1 Center for Genome Science, National Research Institute of Health, KCDC, Cheongju, 28159, Korea; 2 Department of Physiology, School of Medicine, Hanyang University, Seoul, 04763, Korea; 3 Department of Internal Medicine, School of Medicine, Eulji University, Seoul, 01830, Korea

## Abstract

Here, we tested the performance of the mitochondrial DNA copy number (mtDNA-CN) in predicting future type 2 diabetes (n = 1108). We used the baseline clinical data (age, sex, body mass index, waist-to-hip ratio, systolic and diastolic blood pressure) and the mtDNA-CN, hemoglobin A_1c_ (A_1_C) levels and results of oral glucose tolerance test (OGTT) including fasting plasma glucose, 1-hour glucose, and 2-hour glucose levels, to predict future diabetes. We built a prediction model using the baseline data and the diabetes status at biannual follow-up of 8 years. The mean area under curve (AUC) for all follow-ups of the full model including all variables was 0.92 ± 0.04 (mean ± standard deviation), while that of the model excluding the mtDNA-CN was 0.90 ± 0.03. The sensitivity of the f4ull model was much greater than that of the model not including mtDNA-CN: the mean sensitivities of the model with and without mtDNA-CN were 0.60 ± 0.06 and 0.53 ± 0.04, respectively. We found that the mtDNA-CN of peripheral leukocytes is a biomarker that augments the predictive power for future diabetes of A_1_C and OGTT. We believe that these results could provide invaluable information for developing strategies for the management of diabetes.

Diabetes is one of the most prevalent chronic diseases worldwide. In 2011, it was estimated that about 347 million people had diabetes^1^. The prevalence of diabetes is increasing rapidly, and the worldwide prevalence of diabetes in 2030 is expected to be 4.4% or 366 million people^2^. It is well known that diabetes and its complications require life-long management and impose a socioeconomic burden^3^. Therefore, cost-effective management of diabetes is essential.

The duration of hyperglycemia is a critical predictor of unfavorable outcomes, and early detection and treatment is a cost-effective method for reducing or postponing major complications of type 2 diabetes^4^. Moreover, drug and behavioral intervention can prevent the development of type 2 diabetes^5^. For this reason, early detection may be beneficial for preventing and managing type 2 diabetes. Hemoglobin A_1_C (A_1_C) and/or OGTT appear to be biomarkers that allow the successful prediction of the development of future type 2 diabetes.

Several studies have reported on the utility of A_1_C and/or OGTT to predict future type 2 diabetes^6^,^7^,^8^,^9^,^10^. In these studies, the baseline clinical and laboratory information was gathered from cohorts, and information about the onset of type 2 diabetes was collected after several years of follow-up. It has been shown that the A_1_C level is significantly associated with the timing of type 2 diabetes onset^6^,^7^ and that this relationship is valid after adjustment for other clinical variables. Other studies have shown that the baseline A_1_C and/or fasting plasma glucose (FPG) levels can predict future type 2 diabetes^6^,^7^,^8^,^9^,^10^. Although A_1_C and FPG have predictive power for future type 2 diabetes, an additional biomarker that is capable of augmenting the predictive power of the A1C and FPG is needed.

The mitochondrial DNA copy number (mtDNA-CN) is another biomarker for type 2 diabetes. Here, mtDNA-CN indicates the quantity of mitochondria per cell. A low mtDNA-CN precedes the development of type 2 diabetes^11^ and is related to insulin resistance, the age at onset of type 2 diabetes and its complications^12^,^13^,^14^. Based on these previous studies, we hypothesized that the mtDNA-CN has the potential to predict future type 2 diabetes, especially in combination with FPG and A_1_C. To evaluate our hypothesis, we used cohort data that included 8 years of follow-up. Using the cohort data, we tested whether baseline mtDNA-CN has predictive power with or without A_1_C and FPG. For this purpose, we measured the mtDNA-CN at baseline in all participants. Then, using follow-up data, we built and tested the performance of a number of prediction models. We found that including the mtDNA-CN strongly increased the performance of the prediction models for type 2 diabetes, especially in terms of their sensitivity.

## Results

We selected 1,149 blood samples from cohort participants and, after excluding 41 samples because of poor quality, included 1,108 participants in this study. We analyzed the baseline mtDNA-CN and 15 clinical and biochemical variables selected because they are well-known risk factors for diabetes development. The clinical and biochemical characteristics are listed in Table 1. Based on the baseline OGTT results, the sample population was divided into two groups: the nondiabetes group (*n* = 1,005) and diabetes group (*n* = 103).

We performed follow-up investigations of the nondiabetes group every 2 years. Up to the second follow-up, there were no participants lost to follow-up. However, at the third follow-up 141 participants had been lost to follow-up and at the fourth follow-up, another four participants had been lost, meaning that overall, 145 participants were lost to follow-up. During the 8 years of follow-up, type 2 diabetes developed in 142 participants. In the Ansan/Ansung cohort, the number of participants decreased as follow-up proceeded. There were 757 participants that were lost at third follow-up, not at first and second follow-up. Our study population included 141 of the 757 participants. This is the main reason why many participants were lost at third follow-up in our study population. As far as we know, there was no bias for selecting our study population. Our study population seems to contain the 141 participants by chance.

Although the main purpose of this research was to evaluate the predictive power of mtDNA-CN for future diabetes, we tested the differences of all variables between participants with current diabetes and nondiabetes status. Of the glucose metabolism variables, including A_1_C, FPG, 1-h glucose, and 2-h glucose, the 2-hour glucose showed the most significant difference (Table 1). As shown in previous studies, the mtDNA-CN level was significantly lower in the diabetes group: the average log2-transformed mtDNA-CN in the nondiabetes group was 6.82, while that in the diabetes group was 6.51. All other variables except sex and alcohol intake also showed significant differences.

We tested correlation between baseline fasting glucose level and mtDNA-CN level. The Pearson correlation coefficient was −0.08 (*p* value; 5.19 × 10^−3^, confidence interval; −0.15 ~ −0.03) within non-diabetes population and –0.15 (*p* value; 3.85 × 10^−7^, confidence interval; −0.21 ~ −0.09) within total population at baseline investigation. These results were consistent with previous research^15^. Given these, it seems that glucose is negatively correlated with mtDNA-CN level. Although this finding is not enough to determine directionality of effect between glucose and mtDNA-CN, we could use this negative relationship to predict type 2 diabetes.

When we performed a two-group comparison at first follow-up, we found that the 1-h glucose showed the most significant difference (*p* = 6.60 × 10^−20^; Table 2). It was interesting that while the glucose metabolism variables showed lower significance than in the baseline analysis, the significance of mtDNA-CN was greater than in the baseline analysis. This trend also appeared for smoking history and a family history of diabetes. While smoking history and a family history of diabetes showed marginal significance in the baseline data (*p* = 0.21 and 0.09, respectively), they showed substantial significance in the analysis of the first follow-up data (*p* = 5.89 × 10^−5^ and 3.88 × 10^−3^). Sex is another variable that showed significance in the first follow-up data analysis (*p* = 0.02) while showing no significance in the baseline data analysis (*p* = 0.35). In contrast, while age at baseline was significantly different between the diabetes and nondiabetes groups (*p* = 1.77 × 10^−3^), this significance was lost in the analysis of the first and subsequent follow-ups.

In the analysis of the data for the second follow-up, there was a notable trend in the significance of the mtDNA-CN and glucose metabolism variables. Compared with the results of the previous follow-up, the significance of the mtDNA-CN and 1-h glucose increased strikingly: the ratio of the significance of the mtDNA-CN for the first and second follow-ups was 4.58 × 10^10^ (2.97 × 10^−17^/6.48 × 10^−28^), while that of the 1-h glucose was 4.13 × 10^10^ (6.60 × 10^−20^/1.60 × 10^−29^). Although the change was not as great as those for the mtDNA-CN and 1-h glucose, the significance of the A_1_C, FPG, and 2-h glucose also increased substantially: the ratios of significance of A_1_C, FPG, and 2-h glucose for the first and second follow-ups were 5.17 × 10^6^ (8.32 × 10^−12^/1.61 × 10^−18^), 1.04 × 10^4^ (7.71 × 10^−15^/7.41 × 10^−19^) and 1.09 × 10^4^ (2.00 × 10^−12^/1.82 × 10^−16^), respectively. Among the clinical variables, smoking history showed a substantial change of significance between first and second follow-ups (Table 2).

In the data for the third follow-up, mtDNA-CN and the glucose metabolism variables showed higher significance than the other variables (Table 2), although they were less significant than in the results for the second follow-up. Interestingly, A_1_C and hypertension history showed a slight increase in significance from the second follow-up.

The results for mtDNA-CN and glucose metabolism variables in the data from the fourth follow-up maintained the same trend as those from the third follow-up, in that the significance of the variables was lower than for the third follow-up. However, the magnitude of the changes between the third and fourth follow-up was relatively small compared with those between the second and third follow-up. For example, the ratio of the significance of the 1-h glucose between the third and fourth follow-up was 34.0, which was far less than that between previous follow-ups. In contrast, the significance of the WHR and hypertension history increased from that of the third follow-up.

Since we used log-transformed mtDNA-CN values, the differences between mtDNA-CN values seems to be not so distinguishable. However, considering the variance simultaneously, the differences between non-diabetes and diabetes are clear in the box plots (Supplementary Fig. S2). Moreover, the *p* values between nondiabetes and diabetes were highly significant in all follow-ups. These indicates that there were substantial differences of mtDNA-CN between nondiabetes and diabetes in all follow-ups.

When we divided the baseline mtDNA-CN level into 4 categories according to the 25, 50 and 75 percentile values and applied Chi-square test, there were significant differences in prevalence of diabetes between the categories. The *p* value of the Chi-square test between the mtDNA-CN categories and future development of type 2 diabetes at 1 st, 2nd, 3rd and 4th follow-up was 3.40 × 10^−21^, 1.03 × 10^−33^, 2.73 × 10^−17^ and 6.24 × 10^−15^, respectively. Supplementary Table S1 showed that contingency table of the mtDNA-CN categories and type 2 diabetes of all follow-ups. It is clear that the prevalence of type 2 diabetes increases as the percentile of mtDNA-CN decreases.

To assess the performance of the combination of mtDNA-CN and glucose metabolism in the prediction of future diabetes, we applied logistic regression analysis. We used information about the cumulative number of participants with diabetes at each follow-up. For all follow-ups, the overall performance of the prediction models was augmented when the mtDNA-CN was included in the model. In analyzing the results of all follow-ups, we identified two interesting features. The first was that when the mtDNA-CN was included in the model, the sensitivity increased. For example, at second follow-up, the sensitivity increased from 0.55 to 0.68 by adding the mtDNA-CN into the prediction model. The average increase in sensitivity for all follow-ups was 0.73. The second feature was that the overall performance of the prediction model was highest at second follow-up: the AUC of the prediction model at second follow-up increased to 0.96.

We built classification models including different combinations of variables. First, we built a model including the demographic and biometric variables age, sex, BMI, WHR, systolic blood pressure (SBP), and diastolic blood pressure (DBP). This model was designated as model 1 (M1). We constructed an M1 for each follow-up time point. After building the models, we measured their performance in terms of sensitivity, specificity, PPV, NPV, and AUC. Table 3 shows the performance of each model.

The M1s for all follow-ups showed poor performance in terms of sensitivity and PPV, while having high specificity and NPV. Even though the AUC was highest at second follow-up, the sensitivity and NPV was near zero (Table 3). The overall AUC of the M1s for all follow-ups was 0.75 (Table 3). This pattern of performance of the M1 was maintained through to the last follow-up. Among the performance measures, only the PPV improved markedly after the first follow-up: it increased from 0.25 to > 0.50 (Table 3). The overall performance of M1 was highest for the first follow-up (AUC = 0.76).

We built model 2 (M2) by adding A_1_C, FPG, 1-h glucose, and 2-h glucose to the variables included in M1. Compared with M1, the results of M2 showed increased sensitivity and PPV for all follow-up points (Table 3): the average sensitivity for all follow-ups increased from 0.08 to 0.53 and the average PPV also increased from 0.48 to 0.80. Consequently, the average increase in the AUC was 0.15 (from 0.75 to 0.90). M2 showed the highest AUC at the second follow-up (AUC = 0.93).

The performance of model 3 (M3), which was constructed by adding the mtDNA-CN to M2, was better than that of M2, especially for sensitivity and AUC (Table 3). The average AUC of M3 was 0.92, which was 0.02 higher than that of M2 and 0.18 higher than that of M1. The increase in sensitivity was more obvious: the average sensitivity of M3 increased by 0.13 compared with M2 and by 0.47 compared with M1. The other performance measures of the M3 showed no substantial difference to those of M1 and M2.

In addition to these three models, we built another two models and tested their performance. Model 4 (M4) included the variables (A_1_C and FPG) that are available before OGTT is performed, except for the mtDNA-CN. Model 5 (M5) consisted of M4 plus the mtDNA-CN. These models were constructed to test the difference in performance between models with and without the mtDNA-CN in the situation where the 1-h and 2-h glucose are not available. The results demonstrated that M5 outperformed M4. The average AUC of M5 was 0.90, which was 0.04 higher than that of M4 (Table 4). As was the case for the other models, the change in performance was most obvious for sensitivity. The average sensitivity of M5 was 0.52, which was 0.11 higher than that of M4. The other measures of M5 performance were similar to those of M4.

## Discussion

In this analysis, we identified the utility of the mtDNA-CN for predicting the future development of diabetes. The results indicated that the mtDNA-CN has a substantial potential for predicting future diabetes, especially when combined with conventional markers including A_1_C and OGTT results.

The univariate analysis revealed that the mtDNA-CN showed the highest significance of all the variables except 1-h glucose. Although previous studies reported that the mtDNA-CN of people with diabetes is different from that of the normal population, no study has identified whether this difference of mtDNA-CN is more significant than the difference of A_1_C and FPG between the groups, which has been widely investigated for prediction of diabetes. To our knowledge, this is the first study that analyzes the significance for predicting future diabetes by comparing baseline mtDNA-CN, A_1_C, and FPG between nondiabetes and diabetes groups. The significance of the mtDNA-CN in the two-group comparison is the second highest of all the variables that were used in the univariate two-group comparison, and this significance was maintained through all follow-ups. These results indicate that the mtDNA-CN has a huge potential as a biomarker of future diabetes. We also found in the two-group comparison tests that 1-h glucose had the highest significance of the glucose metabolism-related variables, which is consistent with previous findings^16^. In univariate analysis, some variables maintained their significance throughout the follow-up, while the significance of others varied according to the follow-up time. For example, although the mtDNA-CN showed high significance throughout, the magnitude of the significance varied for different follow-up times. The significance of the mtDNA-CN was highest at the second follow-up (*p* = 6.48 × 10^−28^) and decreased as the follow-up time increased. However, the significance of the A_1_C, FPG, 1-h glucose, and 2-h glucose did not vary as much as that of the mtDNA-CN. The other variables also showed less variability during all follow-ups. Interestingly, the significance of a history of hypertension increased as the follow-up proceeded: while its *p* value at the first follow-up was 4.35 × 10^−5^, it decreased to 9.26 × 10^−9^ over time. Given that previous studies showed a relationship between diabetes and hypertension, we confirmed that a history of hypertension can be used as a predictive baseline indicator for future diabetes that will develop in the medium to long term.

It should be noted that we determined the mtDNA-CN of peripheral blood mononuclear cells (PBMC). Organs including pancreas, muscle, liver, and brain are known to participate in glucose homeostasis and the mononuclear cell is not included in the organs that are associated with glucose metabolism. This is a limitation for mtDNA-CN to be applied to biomarker for diabetes directly. However, the mtDNA-CN of PBMC is associated with aberrant glucose metabolism^17^. It is more likely that the difference in mtDNA-CN between nondiabetes and diabetes groups results from hyperglycemia. Palmeira *et al*. reported that the glucose level regulates mtDNA-CN by modulating the transcriptional activity of mitochondrial transcriptional factor A in human liver cancer cell line^18^. They postulated that his modulation is mediated by hyperglycemia-induced production of reactive oxygen species. Moreover, an elevated glucose level is a well-known predictor of future diabetes. A decrease in the mtDNA-CN might reflect hyperglycemia and therefore have the predictive power, which would be synergistic to conventional markers such as glucose and A_1_C. The reason for the higher significance with respect to future diabetes in the two-group comparison of the mtDNA-CN compared with FPG, A_1_C, and 2-h glucose remains unclear, and should be evaluated in further studies.

In the development of our prediction model, we used five different combinations of variables. The performance of each model was correlated with the results of the two-group comparison analysis. M1 comprised clinical variables such as age, BMI, WHR, and blood pressure and the significance of the variables was much lower than in models containing mtDNA-CN and glucose-related variables (FPG, 1-h glucose, 2-h glucose, A_1_C). Consequently, the performance of M1 was the poorest of all the models, especially in terms of sensitivity. For the data from all follow-ups the sensitivity of M1 was almost zero. After inclusion of A_1_C and the results of OGTT, the sensitivity and PPV increased sharply. M2 outperformed M1 in sensitivity, PPV, and AUC. During all follow-ups, the average increase in sensitivity, PPV, and AUC of M2 compared with M1 was 0.53, 0.98, and 0.90, respectively. Similarly to M1, M2 showed the best performance at the second follow-up. M3 showed better performance than M2 for sensitivity and AUC. These results indicated that prediction models including the mtDNA-CN outperformed other models including only conventional predictive markers.

It is clear that the glucose level has predictability for future type 2 diabetes. However, the amount of improvement that were obtained by integrating the mtDNA-CN was relatively smaller than that of glucose levels. Especially, the M2 (without mtDNA-CN) and M3 (with mtDNA-CN) showed only 0.02 difference of AUC in average (Table 3). However, when we applied the roc.test function of pROC package to determine significance of difference between AUCs of M2 and M3, the *p* value was 1.84 × 10^−3^, 2.43 × 10^−4^, 6.74 × 10^−3^ and 1.49 × 10^−2^ at first, second, third and fourth follow-up, respectively. These results indicated that the mtDNA-CN has predictability that could not be obtained by glucose alone.

We built another two models to test the utility of the mtDNA-CN in a single-sampling condition: because obtaining 1-h and 2-h glucose levels can be difficult for patients these two results may not be available, especially in large population-screening settings. Therefore, we evaluated the performance of prediction models containing variables that can be obtained at a single time. When comparing the models with (M5) or without (M4) the mtDNA-CN, the difference between their average sensitivity was obvious. The average sensitivity of M5 was 0.52, which was 0.11 greater than that of M4. The AUC of M5 was also 0.02 greater than that of M4. Both M4 and M5 showed the best performance at the second follow-up. In particular, the AUC of M5 (AUC = 0.93) was equivalent to that of M2 (0.93) that includes FPG, 1-h glucose, and 2-h glucose. We assume that this result clearly indicates the utility of the mtDNA-CN in the prediction of future diabetes.

We performed statistical test for identifying the significance of coefficients of the prediction models whether the mtDNA-CN has independent effect with adjusting the other variables. The results were highly significant, which indicated that the mtDNA-CN has an independent effect (See Supplementary Table S2). Moreover, the *p* values of the mtDNA-CN were the most significant among variables. For example, the coefficient and *p* value of the mtDNA-CN in prediction model of 2^nd^ FU was −51.70 and 6.22 × 10^−12^, which were the largest absolute coefficient value and most significant *p* value among variables. Considering that multiple regression estimates a coefficient of one variable with adjustment of effects of the other variables, it seems that the mtDNA-CN is independent factor for prediction of type 2 diabetes.

It was interesting that the significance of the two-group comparison analysis and the performance of the multivariate prediction model varied according to the follow-up time. In particular, the significance and performance were highest at the second follow-up. The second follow-up occurred approximately 4 years after the baseline investigation. This result implies that the predictive markers and models for future diabetes obtained at baseline are most effective over the subsequent 4 years. Previous studies of the prediction of future diabetes have usually analyzed performance over a fixed period and, therefore, gave no information about the period over which the baseline value of markers could effectively predict future diabetes. Here, we identified that the baseline values of the mtDNA-CN, A_1_C, FPG, and 1-h and 2-h glucose results were most effective predictors within the following 4 years.

In this research, we used mean of the replicate mtDNA-CN values without consideration of variations. This might be biased estimation of mtDNA-CN. Therefore, robust estimation method of mtDNA-CN should be considered for clinical usage.

In conclusion, we demonstrated that the mtDNA-CN of PBMC showed utility for predicting future diabetes. The inclusion of the mtDNA-CN augmented the predictive performance of A_1_C and OGTT results, which are widely studied as conventional predictive markers for future diabetes. We also identified that a predictive model consisting of the baseline values of the markers was most predictive within 4 years of follow-up. We believe that these important results should be applied to the development of strategies for the control of diabetes development.

## Methods

We collected study samples from the Ansan/Ansung cohort, which is an ongoing cohort that was recruited from June, 2001 to January, 2003 and is maintained by the Center for Genome Science of the National Research Institute of Health (NIH), Korea Center for Disease Control & Prevention (KCDC)^19^. All sampling and experiment followed the guidelines provided by Enforcement Decree of Bioethics and Safety Act. The cohort was used in a previous study of the role of the A_1_C level in the screening and prediction of type 2 diabetes^7^. Informed consent has been obtained from all participants and institutional board of National Research Institute of Health, KCDC has given approval. The database for this cohort contains a large amount of clinicoepidemiological information comprising 2,512 variables and 10,000 participants, and gathers follow-up information biannually. Blood samples were collected from the participants at baseline and were deposited in the National Biobank of Korea at the National Institute of Health, KCDC. After sampling of participant’s blood, leukocytes were separated and quality was checked and stored at −10 °C within 4 hours. Then, DNA was extracted within 12 hours and stored at −70 °C. In this research, we used the epidemiological data from the baseline, first, second, third and fourth follow-ups. The follow-ups were done by visiting assigned hospitals. The median length of follow-up was 7.9 years. We quantified the mtDNA-CN in the baseline blood samples.

From the cohort population, we randomly selected 1,108 participants whose initial blood samples were available. The participants had no missing data in their variables used in this analysis. The diagnosis of diabetes was made according to the 1997 America Diabetes Association criteria. After a fast of 8–14 h, all participants underwent a 75 g OGTT. The diagnostic criterion for type 2 diabetes was an FPG concentration ≥7 mmol/L (126 mg/dL) or a 2 h plasma glucose concentration ≥11.1 mmol/L (200 mg/dL). For the follow-up data analysis, we included those participants who had a normal OGTT result at baseline. The follow-up was performed at 2-yearly intervals, and every participant underwent OGTT. During OGTT, we measured blood glucose three times: at the beginning of glucose loading, 1 h after glucose loading and 2 h after glucose loading. The three measurement times were designated as FPG, 1-h glucose, and 2-h glucose, respectively. In the Ansan/Ansung cohort, the drinking status was classified into 3 categories; non-drinker (labeled as 1), past drinker (labeled as 2) and current drinker (labeled as 3). We considered the label 1 participants negative and the rest of the participants positive drinking status. The smoking status was classified into 4 categories; non-smoker (labeled as 1), past smoker (labeled as 2), current occasional smoker (labeled as 3) and current habitual smoker (labeled as 4). The label 1 participants were considered negative and the rest of participants were considered positive smoker.

To determine the mtDNA-CN, the relative copy number of chromosomal segments was measured using real-time quantitative PCR as described previously^20^. SYBR Green-based PCR was performed using the ABI Prism 7900HT sequence detection system (PE Biosystems, Applied Biosystems, Foster City, CA, USA). The target site was ND1 gene of mitochondria. The reference gene was the factor VIII gene, which was used to normalize the input DNA. For each participant, the copy number was determined three times, and the mean copy number was used for analysis (See Supplementary Method for details).

### Statistical analysis

To compare the mtDNA-CN and the other cohort variables between the nondiabetes and diabetes groups, the two-sample *t* test and Fisher’s exact test were used. Since the original mtDNA-CN is count data, we performed log-transformation for application of *t* test. As shown in Supplementary Fig. S1, the original distribution was skewed to left. After log transformation, the shape of histogram was close to normal distribution. The log-transformed were used in all analyses of this research. To determine the predictive power of the mtDNA-CN and other clinical variables for future type 2 diabetes, we applied logistic regression. After model construction, the sensitivity, specificity, positive predictive value (PPV) and negative predictive value (NPV) were estimated for measurement of performance. These computations were repeated for each follow-up period. All computations were performed using R statistical software^21^. AUC and receiver-operating characteristic (ROC) curve were estimated using the ROCR R package^22^. Confidence intervals for the AUC were estimated using the pROC package^15^.

## Additional Information

**How to cite this article**: Cho, S. B. *et al*. Mitochondrial DNA copy number augments performance of A_1_C and oral glucose tolerance testing in the prediction of type 2 diabetes. *Sci. Rep.* **7**, 43203; doi: 10.1038/srep43203 (2017).

**Publisher's note:** Springer Nature remains neutral with regard to jurisdictional claims in published maps and institutional affiliations.

## Supplementary Material

Supplementary Information

## Figures and Tables

**Table 1:** Summary statistics for baseline data.

Variable	NDM (mean ± SD)	DM (mean ± SD)	p value
Glu120 (mmol/L)	5.59 ± 1.39	13.21 ± 3.09	5.28 × 10^−46^
Glu60 (mmol/L)	7.26 ± 2.18	14.08 ± 3.02	7.16 × 10^−43^
A_1_C (%)	5.51 ± 0.37	6.7 ± 1.07	1.23 × 10^−19^
FPG (mmol/L)	4.52 ± 0.44	6.53 ± 1.86	6.24 × 10^−19^
mtDNA-CN	6.82 ± 0.44	6.51 ± 0.38	3.27 × 10^−12^
SBP (mmHg)	120.75 ± 16.87	129.15 ± 17.44	7.83 × 10^−06^
BMI	24.21 ± 2.91	25.67 ± 3.32	3.28 × 10^−05^
WHR	0.89 ± 0.07	0.91 ± 0.06	1.17 × 10^−04^
pHTN (−/+)	881/124	75/28	1.26 × 10^−04^
Age	51.34 ± 8.77	54.95 ± 9.05	1.77 × 10^−4^
DBP (mmHg)	80.6 ± 11.21	84.62 ± 10.40	3.07 × 10^−4^
FDM (−/+)	921/84	88/15	4.48 × 10^−02^
Smoking (−/+)	546/459	47/56	9.76 × 10^−02^
Drink (−/+)	440/565	38/65	2.10 × 10^−01^
Sex (M/F)	556/449	62/41	3.51 × 10^−01^

NDM, non-diabetes; DM, diabetes; SD, standard deviation; Glu60, 1-h glucose; Glu120, 2-h glucose; A1C, hemoglobin A1C; FPG, fasting plasma glucose; SBP, systolic blood pressure; BMI, body mass index; WHR, waist-to-hip ratio; DBP, diastolic blood pressure; FDM, family history of diabetes; pHTN, past history of hypertension.

**Table 2:** Result of the two-group comparison between future diabetes and nondiabetes group.

Variable	First follow-up (n = 1,005)		*p* value	Second follow-up (n = 1,005)		*p* value	Third follow-up (n = 868		*p* value
	NDM (n = 934)	DM (n = 71)		NDM (n = 894)	DM (n = 111)		NDM (n = 740)	DM (n = 128)	
Glu60 (mmol/L)	7.01 ± 1.96	10.51 ± 2.33	6.60 × 10^−20^	6.90 ± 1.90	10.11 ± 2.20	1.60 × 10^−29^	6.91 ± 1.93	9.68 ± 2.39	4.17 × 10^−25^
mtDNA-CN	6.85 ± 0.43	6.4 ± 0.34	2.93 × 10^−17^	6.87 ± 0.42	6.38 ± 0.35	6.48 × 10^−28^	6.86 ± 0.43	6.45 ± 0.38	7.11 × 10^−23^
Glu120 (mmol/L)	5.42 ± 1.15	7.84 ± 2.07	7.71 × 10^−15^	5.38 ± 1.14	7.31 ± 1.88	7.41 × 10^−19^	5.40 ± 1.14	7.03 ± 1.95	5.09 × 10^−16^
FPG (mmol/L)	4.47 ± 0.39	5.10 ± 0.62	2.00 × 10^−12^	4.46 ± 0.38	4.99 ± 0.56	1.82 × 10^−16^	4.46 ± 0.38	4.9 ± 0.59	1.16 × 10^−13^
A_1_C (%)	5.48 ± 0.02	5.96 ± 0.03	8.32 × 10^−12^	5.46 ± 0.31	5.95 ± 0.48	1.61 × 10^−18^	5.45 ± 0.31	5.89 ± 0.47	1.23 × 10^−18^
WHR	0.88 ± 0.07	0.92 ± 0.05	1.91 × 10^−05^	0.88 ± 0.07	0.92 ± 0.06	1.77 × 10^−07^	0.88 ± 0.07	0.91 ± 0.07	5.86 × 10^−06^
DBP (mmHg)	80.20 ± 11.23	85.77 ± 9.65	1.29 × 10^−05^	80.02 ± 11.26	85.24 ± 9.73	5.52 × 10^−07^	80.09 ± 11.13	84.86 ± 11.03	1.18 × 10^−05^
pHTN (−/ + )	831/103	103/21	4.35 × 10^−05^	804/90	77/34	3.29 × 10^−08^	667/73	90/38	2.49 × 10^−08^
SBP (mmHg)	121.11	129.18	7.37 × 10^−05^	119.88 ± 16.6	127.69 ± 17.46	1.64 × 10^−05^	119.86 ± 16.47	127.22 ± 18.11	2.92 × 10^−05^
Smoking (−/ + )	524/410	22/49	5.89 × 10^−05^	506/388	40/71	4.72 × 10^−05^	419/321	51/77	5.17 × 10^−04^
BMI	24.11 ± 2.87	25.42 ± 3.1	1.16 × 10^−03^	24.04 ± 2.82	25.55 ± 3.26	6.95 × 10^−06^	24.07 ± 2.81	25.37 ± 3.30	4.41 × 10^−05^
FDM	865/69	56/15	3.88 × 10^−04^	832/62	89/22	3.58 × 10^−05^	684/56	106/22	1.19 × 10^−03^
Sex (M/F)	507/427	49/22	1.83 × 10^−02^	482/412	74/37	1.13 × 10^−02^	404/336	82/46	5.36 × 10^−02^
Drink (−/ + )	415/519	25/46	1.38 × 10^−01^	402/409	38/73	3.33 × 10^−02^	328/412	45/83	5.39 × 10^−02^
Age	51.24 ± 8.76	52.66 ± 8.90	1.96 × 10^−01^	51.18 ± 8.75	52.57 ± 8.88	1.24 × 10^−01^	50.99 ± 8.42	52.56 ± 9.22	7.28 × 10^−02^
	**Fourth follow-up (n = 864)**								
**Variable**	**NDM (n = 722)**	**DM (n = 142)**	***p* value**	***p* (mean)**					
Glu60 (mmol/L)	6.87 ± 1.89	9.59 ± 2.49	1.61 × 10^−25^	1.65 × 10^−20^					
mtDNA-CN	6.87 ± 0.42	6.48 ± 0.4	4.89 × 10^−21^	7.32 × 10^−18^					
Glu120 (mmol/L)	5.39 ± 1.12	6.85 ± 1.96	6.37 × 10^−15^	3.65 × 10^−15^					
FPG (mmol/L)	4.46 ± 0.38	4.86 ± 0.58	6.67 × 10^−13^	6.96 × 10^−13^					
A_1_C (%)	5.46 ± 0.31	5.86 ± 0.47	4.77 × 10^−18^	2.08 × 10^−12^					
WHR	0.88 ± 0.07	0.91 ± 0.07	5.79 × 10^−07^	6.42 × 10^−06^					
DBP (mmHg)	79.78 ± 10.95	84.82 ± 11.3	1.18 × 10^−05^	9.28 × 10^−06^					
pHTN (−/+)	653/69	101/41	9.26 × 10^−09^	1.09 × 10^−05^					
SBP (mmHg)	119.6 ± 16.32	126.97 ± 17.96	1.01 × 10^−05^	3.24 × 10^−05^					
Smoking (−/+)	414/308	58/84	4.31 × 10^−04^	2.63 × 10^−04^					
BMI	24.03 ± 2.83	25.4 ± 3.27	5.45 × 10^−06^	3.04 × 10^−04^					
FDM	670/52	120/22	2.72 × 10^−03^	1.08 × 10^−03^					
Sex (M/F)	670/52	120/22	2.72 × 10^−03^	2.15 × 10^−02^					
Drink (−/+)	321/401	50/92	5.12 × 10^−02^	6.92 × 10^−02^					
Age	51.24 ± 8.62	52.63 ± 9.07	9.46 × 10^−02^	1.22 × 10^−01^					

NDM, non-diabetes; DM, diabetes; A_1_C, hemoglobin A_1_C; DBP, diastolic blood pressure; FDM, family history of diabetes; FPG, fasting plasma glucose; Glu60, 1-h glucose; Glu120, 2-h glucose; pHTN, past history of hypertension; SBP, systolic blood pressure; SD, standard deviation.

**Table 3:** Performance of prediction analysis with models M1, M2 and M3.

Follow-up	Model	Sensitivity	Specificity	PPV	NPV	AUC (95% CI)
First	M1	0.00	1.00	0.00	0.93	0.76 (0.70–0.82)
	M2	0.59	0.99	0.86	0.97	0.92 (0.89–0.96)
	M3	0.62	0.99	0.85	0.97	0.95 (0.92–0.97)
Second	M1	0.05	0.99	0.50	0.89	0.76 (0.71–0.81)
	M2	0.55	0.98	0.77	0.95	0.93 (0.91–0.96)
	M3	0.68	0.98	0.84	0.96	0.96 (0.94–0.97)
Third	M1	0.08	0.99	0.53	0.86	0.73 (0.68–0.78)
	M2	0.49	0.98	0.79	0.92	0.89 (0.84–0.91)
	M3	0.57	0.98	0.84	0.93	0.90 (0.86–0.93)
Fourth	M1	0.10	0.98	0.56	0.85	0.73 (0.68–0.78)
	M2	0.50	0.98	0.81	0.91	0.87 (0.83–0.90)
	M3	0.55	0.98	0.83	0.92	0.88 (0.85–0.92)
Average	M1	0.06	0.99	0.40	0.88	0.75
	M2	0.53	0.98	0.81	0.94	0.90
	M3	0.61	0.98	0.84	0.95	0.92

NPV, negative predictive value; PPV, positive predictive value; AUC, area under curve.

**Table 4:** Result of prediction analysis with models M4 and M5.

Follow-up	Model	Sensitivity	Specificity	PPV	NPV	AUC (95% CI)
First	M4	0.38	0.99	0.73	0.96	0.88 (0.83–0.93)
	M5	0.41	0.99	0.71	0.96	0.92 (0.88–0.96)
Second	M4	0.44	0.99	0.79	0.93	0.88 (0. 84–0.92)
	M5	0.63	0.98	0.81	0.96	0.93 (0.90–0.96)
Third	M4	0.42	0.98	0.77	0.91	0.84 (0.80–0.88)
	M5	0.55	0.98	0.81	0.93	0.88 (0.84–0.91)
Fourth	M4	0.39	0.98	0.79	0.89	0.82 (0.78–0.86)
	M5	0.50	0.97	0.79	0.91	0.86 (0.82–0.89)
Average	M4	0.41	0.98	0.77	0.92	0.86
	M5	0.52	0.98	0.78	0.94	0.90

NPV, negative predictive value; PPV, positive predictive value; AUC, area under curve.
