# Supplementary material for: Mitochondrial DNA copy number augments performance of A1C and oral glucose tolerance testing in the prediction of type 2 diabetes
Source: Sci Rep. 2017 Mar 2;7:43203. doi: 10.1038/srep43203 (PMC5333082; doi:10.1038/srep43203)

Mitochondrial DNA copy number augments performance of A_1C and oral glucose tolerance testing in the prediction of diabetes

Seong Beom Cho[1,†], InSong Koh[2,†], Hye-Young Nam[1], Jae-Pil Jeon[1], Hong Kyu Lee[3], and Bok-Ghee Han[1,*]

[1]Center for Genome Science, National Research Institute of Health, KCDC, Cheongju, 28159, Korea

[2]Department of Physiology, School of Medicine, Hanyang University, Seoul, 04763, Korea

[3]Department of Internal Medicine, School of Medicine, Eulji University, Seoul, 01830, Korea

Supplementary Method

Quantification of mitochondrial DNA copy number

To determine relative mitochondrial DNA copy number (mtDNA-CN) of peripheral leukocyte, we applied polymerase chain reaction (PCR) using SYBR green. We used factor VIII gene as a reference we estimated mtDNA-CN using difference of threshold cycles (Ct value) between factor VIII and mitochondrial gene ND1. We selected MT-ND1 region because it has no homology with any nuclear DNA sequences in a BLAST search (Ref 16). The primer sequences of the two genes is as follows

Factor VIII	Forward	5'-TACCATCCAGGCTGAGGTTTAT-3'
	Reverse	5'-AAAGAGTTGTAACGCCACCATT-3'
ND1	Forward	5'-ACTACAACCCTTCGCTGACG-3'
	Reverse	5'-GCCTAGGTTGAGGTTGACCA-3'

The PCR was repeated 3 times. At each time, the PCR was repeated twice. In total, 6 times of PCR on the two genes (factor VII and ND1) were performed for each sample. The reaction master mixture was composed of each primer 8uM, 2X SYBR Green PCR master mix and genomic DNA 50 ng. Initial denaturation time at 95 C was 10 minutes. Then, 40 times of thermocycling (95 C for 15 seconds and 60 C for 1 minute per cycle) was performed. The ABI Prism 7900HT sequence detection system (PE Applied Biosystems, Foster City, CA) was used for PCR.

Supplementary Tables

Supplementary Table S1. Prevalence of type 2 diabetes according to the percentiles of mtDNA-CN

**mtDNA-CN category**	**1st FU**		**2nd FU**		**3rd FU**		**4th FU**	
	NDM	DM	NDM	DM	NDM	DM	NDM	DM
**< 25P**	199	52	170	81	153	47	144	48
**25P ~ 50P**	241	13	233	21	185	16	181	22
**50P ~ 75P**	247	3	243	6	209	5	205	6
**>75P**	244	3	243	4	203	3	190	6

P; percentile, FU; follow-up

Supplementary Table S2. Size of coefficients and *p* value variables in the prediction models

	1st FU		2nd FU		3rd FU		4th FU	
	Coef	*p* value	Coef	*p* value	Coef	*p* value	Coef	*p* value
**SBP**	0.0036	0.84	−0.01	0.43	−0.01	0.34	−0.01	0.30
**DBP**	0.02	0.47	0.04	0.09	0.03	0.07	0.04	0.03
**Sex**	−0.15	0.79	0.22	0.68	0.28	0.52	0.33	0.41
**Age**	0.0059	0.83	−0.01	0.53	−0.003	0.84	−0.01	0.62
**A1C**	23.15	9.01×10^{-3}	43.51	2.57×10^{-8}	37.67	7.14×10^{-8}	32.60	4.03×10^{-7}
**FPG**	1.63	3.29×10^{-4}	1.68	6.75×10^{-5}	1.16	1.27×10^{-3}	0.75	0.02
**GLU60**	0.23	0.02	0.32	1.10×10^{-3}	0.23	8.43×10^{-4}	0.32	1.09×10^{-6}
**GLU120**	0.75	1.57×10^{-7}	0.52	4.18×10^{-5}	0.34	8.83×10^{-4}	0.23	0.02
**mtDNA-CN**	−34.68	1.15×10^{-5}	−51.70	6.22×10^{-12}	−35.32	1.70×10^{-9}	−31.36	1.52×10^{-8}
**WHR**	−3.57	0.29	−0.85	0.76	−2.72	0.25	−1.35	0.54
**BMI**	0.04	0.57	0.08	0.16	0.07	0.19	0.08	0.07
**HTN**	0.09	0.85	0.58	0.19	0.76	0.04	0.67	0.05
**DRINK**	−0.30	0.51	0.34	0.39	0.33	0.33	0.38	0.23
**SMOKE**	0.55	0.32	0.27	0.58	0.23	0.56	0.15	0.69
**FmDm**	0.58	0.28	0.79	0.09	0.34	0.42	0.09	0.82

Coef: coefficient, SBP; systolic blood pressure, DBP; diastolic blood pressure, A1C; hemoglobin A1C, GLU60; 1 hour glucose, GLU120; 2 hour glucose, mtDNA-CN; mitochondrial copy number, WHR; waist hip ratio, BMI; body mass index, HTN; hypertension, FmDm; family history of diabetes.

Supplementary Figure Legend

Supplementary Figure S1. Histogram of original and log-transformed mtDNA-CN

Supplementary Figure S2. Boxplot of log-transformed mtDNA-CN in nondiabetes and diabetes group of all follow-ups.

Supplementary Figures

Supplementary Figure S1

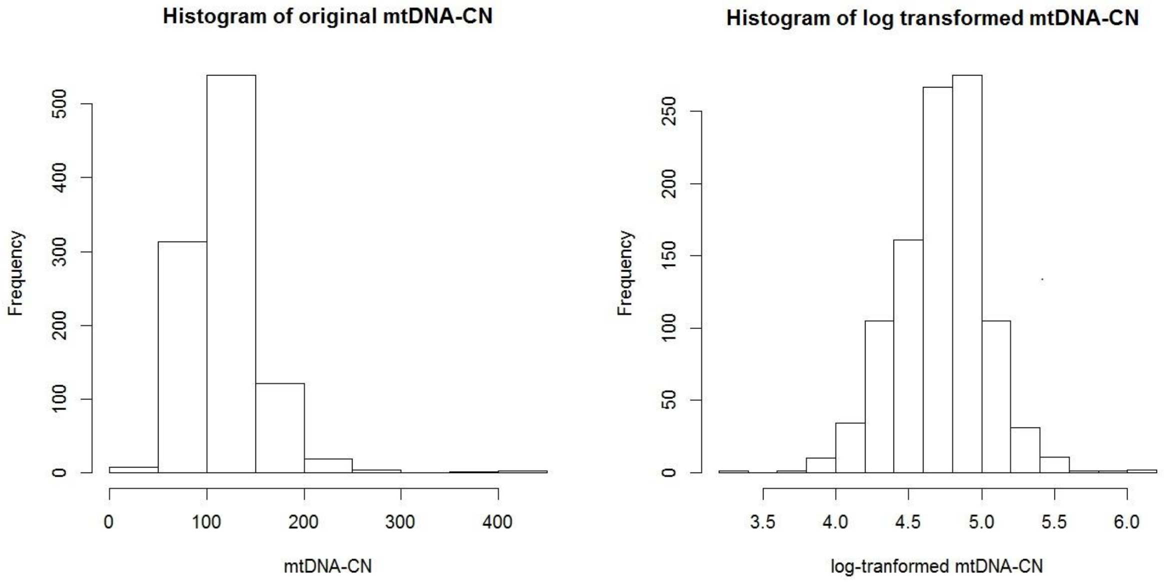

Supplementary Figure S2

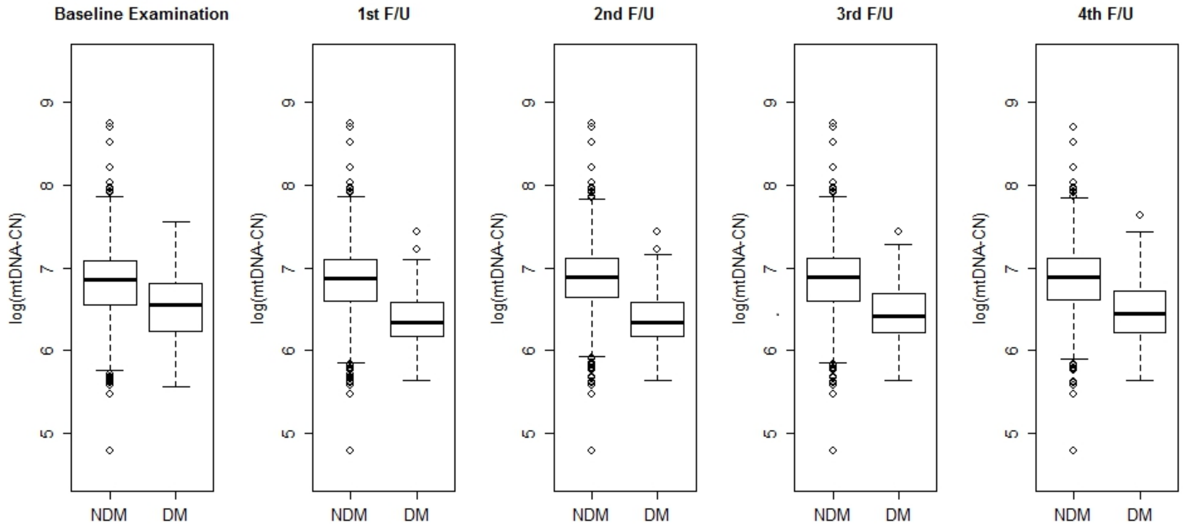

Supplement: Supplementary Information [file srep43203-s1.pdf]
